# Laryngeal Transcriptomic Insights into Echolocation Call Frequency Divergence in Closely Related *Rhinolophus* Species

**DOI:** 10.3390/biology15070548

**Published:** 2026-03-30

**Authors:** Guiyin Miao, Jinhua Cong, Jinhong Lei, Sirui Quan, Jiqian Li, Yannan Li, Kangkang Zhang, Tong Liu

**Affiliations:** 1 College of Life Sciences, Jilin Agricultural University, Changchun 130118, China; 2 Jilin Provincial International Cooperation Key Laboratory for Biological Control of Agricultural Pests, Changchun 130118, China; 3 College of Animal Science and Technology, Jilin Agricultural University, Changchun 130118, China; 4 Jilin Provincial Key Laboratory of Animal Resource and Ecological Security, Northeast Normal University, Changchun 130117, China

**Keywords:** acoustic divergence, closely related species, larynx, RNA-seq, Chiroptera

## Abstract

Acoustic divergence is widely recognized as a key driver of speciation and niche differentiation in vocal animals. In echolocating horseshoe bats (*Rhinolophus*), the larynx is specialized for producing high-duty-cycle signals used in foraging, navigation, and species recognition. While the ecological role of echolocation is established, the molecular mechanisms regulating laryngeal frequency remain unclear. We compared the laryngeal transcriptomes of three closely related, sympatric *Rhinolophus* species with distinct resting frequencies (RFs): *R. episcopus* (~46 kHz), *R. siamensis* (~66 kHz), and *R. osgoodi* (~85 kHz). This comparison identified 511 differentially expressed genes. High-frequency species upregulated genes involved in cytoskeletal dynamics and muscle contraction, such as cell adhesion molecules and motor proteins, while low-frequency species upregulated genes related to cellular homeostasis and metabolic maintenance. Weighted gene co-expression network analysis revealed two RF-correlated modules: a high-frequency module enriched in aerobic respiration and carbon metabolism and a low-frequency module enriched in lipid metabolism. Protein–protein interaction analysis identified *ACTC1*, vital for muscle contraction, as a hub gene. Evolutionary analysis showed that *ACTC1* is highly conserved, with no significant positive selection, indicating that transcriptional regulation, rather than coding-sequence divergence, is the primary driver of the observed functional differences. These findings suggest that RF variation likely results from transcriptional remodeling in laryngeal superfast muscles. This study provides the first transcriptomic evidence linking laryngeal gene expression with acoustic divergence and offers new insights into the genetic basis of bat echolocation.

## Simple Summary

Acoustic divergence drives bat diversification, yet the molecular mechanisms regulating the laryngeal apparatus to produce distinct echolocation signals remain largely unknown. We investigated three closely related, sympatric *Rhinolophus* species that show marked variation in call frequency. Comparative transcriptomics of laryngeal tissue revealed that species with different frequencies use distinct biological pathways, especially in muscle contraction and substrate-specific energy metabolism. Evolutionary analysis of the conserved muscle gene *ACTC1* indicates that these physiological changes are likely due to transcriptional regulation rather than coding sequence mutations. Our findings provide new transcriptomic insights into the physiological adaptations underlying interspecific frequency divergence, offering a fresh perspective on understanding the diversity of bat acoustic signals.

## 1. Introduction

Vocalization is widespread and fundamental across diverse animal taxa, including insects, frogs, birds, and mammals [1,2]. Beyond its role in mediating social interactions, acoustic divergence also acts as a potent driver of ecological niche differentiation and speciation [3]. Even without distinct morphological traits, divergent acoustic signals can enforce species boundaries through behavioral isolation [2]. For example, empirical studies show that differences in call frequency facilitate mate recognition in crickets [4] and tree frogs [5]. Similarly, variation in bird song correlates with both mate selection and niche partitioning [6]. In mammals such as the genus *Microcebus* [7], interspecific acoustic variation serves as an effective mechanism for reproductive isolation under sympatric conditions. Therefore, understanding how acoustic divergence arises is important for revealing the origins and maintenance of biodiversity.

Chiroptera is one of the most widely distributed and evolutionarily successful mammalian orders. It includes more than 1400 recognized species across 21 families and 220 genera [8]. Bats have evolved true powered flight [9] and sophisticated echolocation systems [10,11], which are critical for foraging, navigation, and species recognition [12,13,14]. Driven by these fundamental functions, bat echolocation has undergone profound acoustic divergence, shaped by natural selection, phylogenetic inertia, and morphological constraints [15]. In some species, call features such as frequency and bandwidth adapt to distinct ecological niches and perceptual challenges, often overriding phylogenetic constraints [16]. Additionally, sympatric interactions promote acoustic divergence to minimize interspecific overlap and ensure precise conspecific recognition [17].

Echolocation involves complex processes, including sound production, auditory reception, and neural processing [18,19]. While extensive research focuses on signal detection and processing [20,21,22,23,24,25], the mechanisms of laryngeal sound production remain less explored. Most studies in this area have addressed anatomical structure, biomechanics, and muscle physiology. Evidence indicates that the cricothyroid muscle plays a pivotal role in rapidly altering vocal fold tension to regulate emission frequency, which is essential for high-frequency echolocation [26]. Notably, this tissue is characterized as a “superfast muscle” with extraordinary contraction kinetics [26,27]. Moreover, these properties are supported by a specialized sarcoplasmic reticulum and a high mitochondrial content [27,28], enabling rapid Ca^2+^ cycling and sustained energy. Molecular studies reveal key adaptations in the bat larynx, such as parallel amino acid substitutions in genes related to calcium cycling, sarcoplasmic reticulum, and myosin isoforms [29]. Despite these advances, the specific laryngeal molecules and regulatory pathways underlying interspecific acoustic divergence in bats remain unclear.

Horseshoe bats (genus *Rhinolophus*) represent an ideal model for studying the molecular basis of acoustic divergence among species. Evolutionarily, *Rhinolophus* bats have undergone adaptive radiation, producing many closely related species with distinct echolocation frequencies [30]. Research shows that frequency variations are related to prey choice, habitats, phylogenetic relationships, and physical traits [17,31,32,33,34,35]. Acoustically, these bats emit long-pulse, high-duty-cycle (HDC) echolocation calls with a prominent constant-frequency (CF) component. Importantly, their documented Doppler-shift compensation (DSC) demonstrates precise vocal control [36]. Furthermore, the resting frequency (RF) of the CF component is very stable within species but varies clearly between species [37,38], making it a practical measure of acoustic divergence. These capabilities rely on a specialized laryngeal structure. Specifically, horseshoe bats have a short, strong larynx [39,40,41] with well-developed thyroarytenoid and cricothyroid muscles covering the ventral surface, enabling quick and fine control of vocal fold tension [40]. This system is reinforced by strong cartilage, especially at the top of the cricoid cartilage, providing firm attachment for muscles [39,40,42]. Additionally, horseshoe bats also have tracheal chambers [43], all of which support efficient sound production. Given that HDC echolocation requires rapid muscle contractions and high energy expenditure, we hypothesize that differences in echolocation frequency across species are driven by changes in molecular pathways that regulate muscle contraction and energy use.

To test this hypothesis and provide new insights into acoustic divergence, this study focuses on three closely related *Rhinolophus* species exhibiting significant RF gradients: *R. episcopus* (~46 kHz), *R. siamensis* (~66 kHz), and *R. osgoodi* (~85 kHz). Their co-occurrence in the same cave helps minimize the confounding effects of environmental variation on gene expression. A systematic comparative transcriptomic analysis of their laryngeal tissues tested three hypotheses: (1) higher-frequency calls require rapid laryngeal muscle contraction, reflected in muscle- and calcium-handling gene expression patterns; (2) varying energetic demands of frequency are matched by species-specific metabolic adaptations; and (3) key regulatory genes underlying acoustic adaptations show positive selection signatures. By investigating transcriptional regulation in their laryngeal vocal apparatus, this study provides new evidence on the evolution of echolocation diversity among closely related bats.

## 2. Materials and Methods

### 2.1. Field Sampling and Phenotypic Analysis

Fieldwork was conducted at Jiumen Cave in Lengshuijiang City, Hunan Province, China, in August 2024. Adult male individuals of three sympatric bat species, *Rhinolophus episcopus* (Rhep), *R. siamensis* (Rhsi), and *R. osgoodi* (Rhos), were captured using mist nets as they returned to their roosts.

Echolocation calls were recorded in a temporary field laboratory lined with acoustic foam to minimize environmental noise and echoes. During the recordings, the ambient temperature was maintained at 29 °C and the relative humidity at 80%. To avoid frequency variations caused by DSC during flight, we recorded the calls of single individuals hanging freely within a light mesh bag. Vocalizations from these resting individuals were captured using an Avisoft UltraSoundGate system (Avisoft Bioacoustics, Glienicke/Nordbahn, Germany). The microphone was positioned 50 cm from the bat. Acoustic signals were digitized at a sampling rate of 375 kHz with a 16-bit resolution. For each bat, ten high-quality calls were manually selected. The RF of each call was measured using Avisoft-SASLab Pro software v5.3.2-57 (FFT length: 512 points, 93.75% overlap, spectral resolution: 586 Hz).

In addition, external morphological parameters related to vocalization were measured for each individual. Body weight (W) was measured using an electronic balance to the nearest 0.01 g. Forearm length (FA), sella width (SW), sella height (SH), and horseshoe width (HW) were measured using digital calipers to the nearest 0.01 mm. To minimize the confounding effect of body size, the Body Condition Index (BCI) was calculated as the ratio of body weight to forearm length. To assess the relationships between morphological traits (FA, BCI, SW, SH, HW) and acoustic frequency (RF) among the three *Rhinolophus* species, a correlation matrix was constructed using the GGally package in R v4.4.0 [44].

### 2.2. Tissue Collection and RNA Extraction

After collecting acoustic and morphological data, bats were placed in an induction chamber and anesthetized with 5% isoflurane to minimize suffering. Once death was confirmed by cessation of respiration and heartbeat [45,46], laryngeal tissues were dissected within 5 min, cleared of connective tissue, and submerged in RNAlater (Beyotime Biotechnology, Shanghai, China) at 4 °C overnight before being stored at −80 °C. The entire dissections were performed on ice to suppress RNase activity and prevent post-mortem RNA degradation. Nine individuals (3 per species) were sampled for sufficient biological replication. All procedures were approved by the Animal Ethics Committee of Jilin Agricultural University and conducted in accordance with relevant laws and regulations governing wildlife research in China.

Total RNA was extracted from laryngeal tissues using TRIzol™ reagent (Thermo Fisher Scientific, Waltham, MA, USA). The concentration and purity of RNA were checked using a NanoDrop^®^ 2000 spectrophotometer (Thermo Fisher Scientific, Waltham, MA, USA), with all samples exhibiting OD_260_/_280_ ratios between 1.8 and 2.0. RNA integrity was then confirmed on an Agilent 2100 Bioanalyzer (Santa Clara, CA, USA), with all samples showing RIN > 7.0. Following this quality assessment, high-quality RNA was sent to Novogene Co., Ltd. (Beijing, China) for library construction and sequencing.

### 2.3. Library Preparation and Sequencing

Libraries were constructed using the NEBNext Ultra RNA Library Prep Kit for Illumina (NEB, Ipswich, MA, USA). First, mRNA was enriched from total RNA using oligo (dT) magnetic beads. Fragmentation was carried out using divalent cations at 94 °C for 15 min. First-strand cDNA was synthesized using random hexamer primers and M-MuLV Reverse Transcriptase. Second-strand synthesis was performed using DNA Polymerase I and RNase H. The double-stranded cDNA was then end-repaired, A-tailed, and ligated to sequencing adapters. Next, the ligation products were size-selected and amplified by PCR for 12 cycles. The constructed libraries were then purified to complete the process.

To ensure the reliability of the sequencing data, several library quality control steps were performed. First, initial quantification was done using a Qubit 2.0 Fluorometer (Thermo Fisher Scientific, Waltham, MA, USA). Next, the insert size distribution was analyzed with an Agilent 2100 Bioanalyzer, confirming an average insert size of 200–300 bp. The effective library concentration was then quantified by qPCR (cutoff >1.5 nM). Finally, the qualified libraries were sequenced on the Illumina NovaSeq 6000 platform (San Diego, CA, USA) to generate 150 bp paired-end reads.

### 2.4. Data Quality Control and Quantification

Raw reads were processed with FastQC v0.12.1 [47] to assess sequencing quality, GC content, and adapter contamination. To ensure high-quality data for downstream analysis, raw reads were filtered with Trimmomatic v0.39 [48]. This process removed adapter sequences, low-quality bases, and reads shorter than 50 bp, yielding high-quality clean data.

Given the lack of species-specific reference genomes for the three studied bats, the high-quality genome of the closely related greater horseshoe bat (*R. ferrumequinum*; RefSeq No. GCF_004115265.2) was used. Transcript quantification was performed using Salmon v1.10.3 [49] in quasi-mapping mode. A transcriptome index was built from reference cDNA sequences derived from *R. ferrumequinum*. Clean reads were then pseudo-aligned to this index to estimate transcript-level abundances, which were aggregated into gene-level count matrices using the tximport [50] package in R, based on a transcript-to-gene mapping file. Gene expression levels were normalized as Transcripts Per Million (TPM). Detailed statistics, including raw/clean read counts and mapping rates, are summarized in Table S1.

### 2.5. Differential Expression and Enrichment Analysis

To evaluate the overall variance in gene expression profiles among samples, principal component analysis (PCA) was performed on the regularized log-transformed (rlog) expression matrix. The sample distribution and clustering patterns were visualized using the FactoMineR [51] and ggord [52] packages in R. In addition, pairwise Pearson correlation coefficients among all samples were calculated with the rstatix [53] package using log_2_-transformed, normalized counts, and the resulting correlations were visualized as heatmaps using the pheatmap [54] package in R.

Differential expression analysis was conducted using DESeq2 [55] on gene-level count matrices. Pairwise comparisons were performed for the three species: Rhos vs. Rhep, Rhsi vs. Rhep, and Rhos vs. Rhsi. In each comparison, the latter species served as the reference control. Differentially expressed genes (DEGs) were identified with a threshold of |log_2_FoldChange| ≥ 1 and Benjamini–Hochberg adjusted *p*-value < 0.05. Genes with log_2_FoldChange > 0 were classified as upregulated; those with log_2_FoldChange < 0 were classified as downregulated. The dispersion plot was generated using the empirical Bayes shrinkage method to assess the fit of the negative binomial model. Volcano plots were generated to visualize the DEGs using the tidyverse [56], dplyr [57], and ggplot2 [58] packages in R.

To elucidate the biological functions and pathways associated with the DEGs, gene ontology (GO) [59] functional annotation and Kyoto Encyclopedia of Genes and Genomes (KEGG) [60] pathway enrichment analyses were performed using the clusterProfiler package [61] in R. A significance threshold of *p*-value < 0.05 was applied to identify enriched terms. Additionally, gene set enrichment analysis (GSEA) based on the KEGG database was conducted using the WebGestalt platform [62]. The same significance cutoff (*p*-value < 0.05) was used.

### 2.6. Weighted Gene Co-Expression Network Analysis

To explore the relationship between gene co-expression patterns and phenotypic traits, we conducted a weighted gene co-expression network analysis (WGCNA) using the WGCNA package [63] in R. Before network construction, we transformed gene expression data using log_2_(counts+1) to reduce variation in sequencing depth. Next, highly variable genes were selected using the median absolute deviation (MAD) method, applying a threshold of MAD ≥ 0.08. The weighted adjacency matrix was calculated based on Pearson correlation coefficients for all gene pairs. To satisfy the scale-free topology criterion, the optimal soft-thresholding power was evaluated using the pickSoftThreshold function; however, given our small sample size (*n* = 9) and the scale-free topology fit index (*R*^2^) not reaching 0.85, we chose an empirical power of *β* = 18 for a signed network. This approach prioritized biological directionality and reduced noise from small datasets. The adjacency matrix was then transformed into a topological overlap matrix (TOM), and the corresponding dissimilarity (1-TOM) was used for hierarchical clustering.

Co-expression modules were identified using the dynamic tree cut algorithm. To mitigate instability in module detection due to the limited sample size, we conservatively optimized clustering parameters by setting the minimum module size to 50 and the tree-splitting sensitivity (deepSplit) to 2. To further reduce redundancy, we merged closely related modules with highly correlated module eigengenes (MEs) using a cut height threshold of 0.35. Finally, to identify biologically relevant modules, MEs were correlated with echolocation frequency (RF) and morphological phenotypes (BCI, FA, HW, SW, and SH). Modules exhibiting a significant correlation with RF (|*r*| ≥ 0.3 and *p* < 0.05) were then selected for functional enrichment analysis, as described above.

### 2.7. Protein–Protein Interaction Network and Evolutionary Analysis

To explore potential molecular mechanisms underlying laryngeal muscle kinetics, candidate genes were prioritized from the identified DEGs based on their functional relevance to muscle contraction, cytoskeletal organization, and motor activity. Subsequently, a Protein–Protein Interaction (PPI) network was generated using the STRING database to identify key hub genes, with a strict confidence threshold of ≥ 0.9. The hub gene exhibiting the highest connectivity was selected for downstream molecular evolutionary analysis.

To validate the sequences of the top hub gene, gene-specific primers were designed using NCBI Primer-BLASTv2.5.0 based on the reference genome (Table S2). The coding sequences (CDSs) of the three *Rhinolophus* species were then amplified by PCR and sequenced using the Sanger method (Sangon Biotech, Shanghai, China). Assembly and alignment of these newly sequenced *Rhinolophus* CDS were performed in Geneious v8.1.8. Subsequently, orthologous CDS sequences from 24 representative mammalian species were retrieved from the NCBI database and combined with the newly obtained *Rhinolophus* sequences (Table S3). A species tree was constructed based on established phylogenetic relationships [64,65], while a gene tree was inferred from the hub gene CDS sequences using the Maximum Likelihood (ML) method in MEGA v11 [66]. The GTR+G+I model was identified as the best-fit nucleotide substitution model, and nodal support was assessed with 1000 bootstrap replicates.

To detect signatures of positive selection acting on this hub gene, we implemented the branch-site model using the codeml program in PAML v4 [67]. In separate analyses, the lineages of *R. osgoodi*, the *Rhinolophus* genus, and all echolocating bats were each designated as the foreground branch. We tested for positive selection (ω > 1) by comparing the alternative model to the null model and evaluated statistical significance using likelihood-ratio tests (LRTs) based on a chi-square distribution (*df* = 1) to identify potential adaptive evolutionary changes.

## 3. Results

### 3.1. Phenotypic Variation and Transcriptomic Data

**Figure 1:**
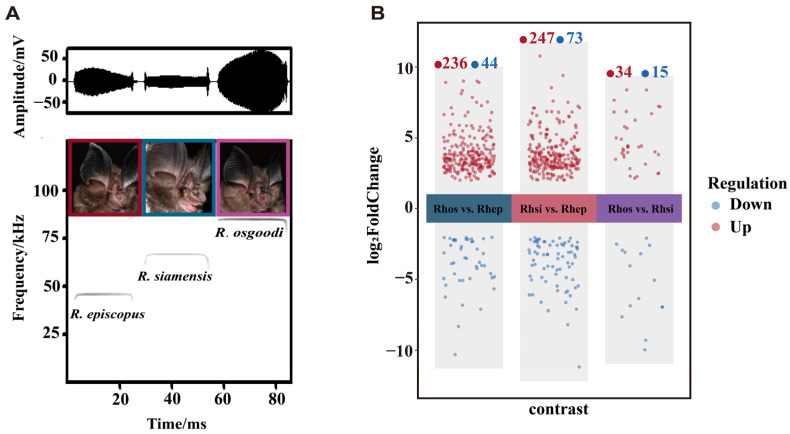
Phenotypic characterization and transcriptomic comparison of the three *Rhinolophus* species. (**A**) Spectrograms and oscillograms of echolocation calls emitted by *R. episcopus*, *R. siamensis*, and *R. osgoodi*, alongside their noseleaf morphology. (**B**) Jitter plot showing the number and distribution of differentially expressed genes (DEGs) in three pairwise comparisons. Red dots indicate upregulated (log2FoldChange > 0) DEGs and blue dots indicate downregulated (log2FoldChange < 0) DEGs, respectively. The counts of DEGs are labeled above each comparison group.

Phenotypic characterization revealed distinct morphological and acoustic divergence among the three species (Figure 1A and Figure S1, Table S1). The largest species, *R. episcopus*, had a significantly greater forearm (FA: 47.13 ± 1.00 mm) and noseleaf size than the other two species, which inversely corresponded to the lowest echolocation frequency (RF: 45.7 ± 0.98 kHz). *Rhinolophus siamensis* and *R. osgoodi* shared comparable body sizes (FA: 39.37 ± 0.79 mm and 39.70 ± 2.46 mm) but differed in acoustic and noseleaf traits. *Rhinolophus siamensis* had an intermediate noseleaf and RF (66.2 ± 0.98 kHz), while *R. osgoodi* possessed the smallest noseleaf and the highest frequency (RF: 85.07 ± 0.58 kHz). Statistical analysis confirmed a significant negative correlation between RF and morphological traits, especially FA and SW (Figure S1).

Transcriptome sequencing of the nine laryngeal samples generated a total of 221,968,027 raw paired-end reads, ranging from 23,338,866 to 29,632,814 reads per sample. After quality control, 218,425,531 high-quality clean reads were retained, with mapping rates ranging from 62.83% to 75.32% across all samples (Table S1).

### 3.2. Identification of Differentially Expressed Genes and Functional Enrichment

Principal component analysis showed a clear separation between the low-frequency *R. episcopus* and the high-frequency species (*R. siamensis* and *R. osgoodi*) along the first principal component (PC1: 23.0%), while the latter two species exhibited overlapping expression patterns (Figure S2A). Pearson correlation matrices revealed high biological reproducibility among replicates, with intra-group correlation coefficients exceeding 0.85 for all species (overall range: 0.82–1.00; Figure S2B). The trend in the dispersion estimation plot confirmed that gene-wise variability was appropriately modeled, ensuring the statistical reliability of the identified DEGs (Figure S3). Comparisons involving *R. episcopus* yielded the highest number of significant DEGs: 280 in Rhos vs. Rhep and 320 in Rhsi vs. Rhep. By contrast, the comparison between the two high-frequency species (Rhos vs. Rhsi) yielded only 49 DEGs, indicating a high degree of genetic similarity between them.

**Figure 2:**
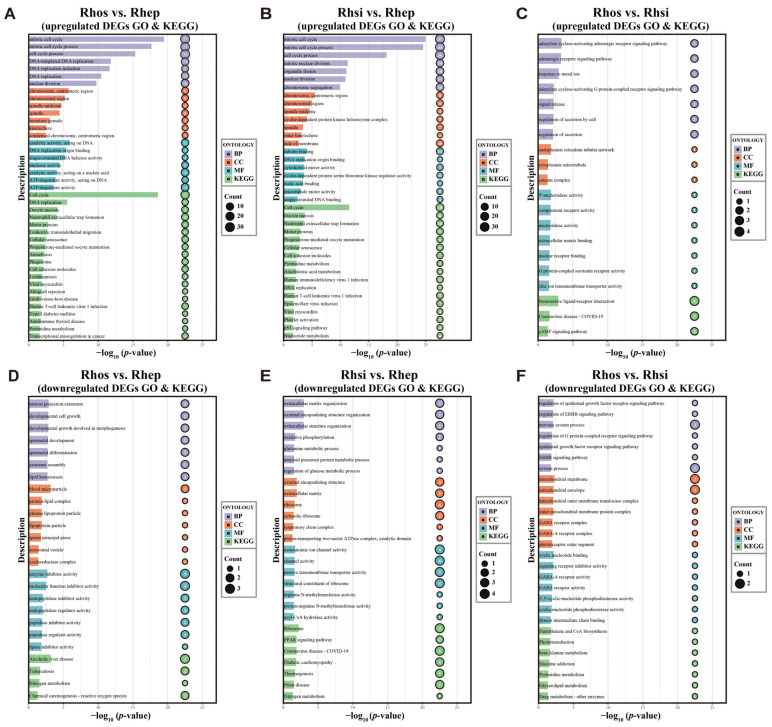
GO and KEGG enrichment analyses of DEGs from three pairwise comparisons of *Rhinolophus* species. (**A**–**C**) GO and KEGG enrichment analysis of upregulated genes. (**D**–**F**) GO and KEGG enrichment analysis of downregulated genes. The y-axis displays the GO/KEGG term descriptions and the x-axis represents the statistical significance as –log_10_(*p*-value). Bubble size corresponds to the number of genes enriched in each term. The top seven GO terms per category and the top 20 enriched KEGG pathways are displayed.

Functional enrichment analysis of upregulated genes revealed strong convergence in structural and proliferative pathways (Figure 2A–C, Tables S4 and S5). Specifically, in both comparisons against *R. episcopus* (Rhos vs. Rhep and Rhsi vs. Rhep), upregulated DEGs were significantly enriched in GO terms associated with cell proliferation and cytoskeletal dynamics, including “Mitotic cell cycle,” “DNA replication,” and “Chromosome, centromeric region.” Consistently, KEGG pathway analysis identified “Cell cycle,” “Motor proteins,” and “Cell adhesion molecules” as key enriched pathways, suggesting a systematic enhancement in cellular turnover and cytoskeletal motility in high-frequency species. In contrast, the comparison between the two high-frequency species (Rhos vs. Rhsi) reflected differences in fine-tuned physiological regulation rather than structural formation. In this case, upregulated genes were primarily enriched in signal transduction and transport mechanisms, such as “Adrenergic receptor signaling,” “Zinc ion transmembrane transporter activity,” “cAMP signaling pathway,” and “Neuroactive ligand–receptor interaction.”

Distinct functional patterns for downregulated genes emerged across comparisons, highlighting species-specific metabolic and homeostatic strategies (Figure 2D–F, Tables S4 and S5). In the Rhos vs. Rhep comparison, downregulated DEGs were associated with cellular homeostasis and structural maintenance, including “Neuron projection extension,” “Developmental cell growth,” “Lipid homeostasis,” and “Enzyme inhibitor activity.” Conversely, the Rhsi vs. Rhep comparison showed downregulation in energy metabolism and protein synthesis, with enrichment in “Oxidative phosphorylation,” “Regulation of glucose metabolic process,” “Glutamine metabolic process,” and “Respiratory chain complex.” KEGG analysis also highlighted the “Ribosome” and “PPAR signaling pathway.” Differentiation between the high-frequency species (Rhos vs. Rhsi) involved downregulated neuro-modulatory and metabolic pathways, including “Nervous system process,” “GABA receptor activity,” and “Glycerolipid metabolism.”

Complementing the KEGG results, GSEA further revealed specific upregulation of neurotransmission-related pathways in the highest-frequency species (*R. osgoodi*), alongside a consistent downregulation of fatty acid metabolism in both high-frequency species (Figure S4).

### 3.3. Co-Expression Modules Correlated with Echolocation Frequency

**Figure 3:**
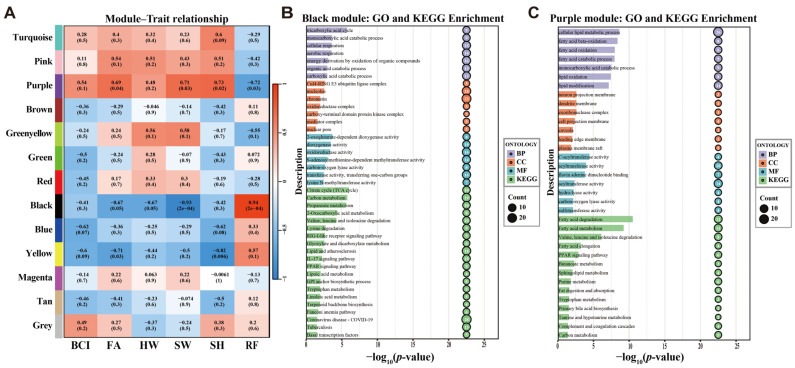
Co-expression modules associated with phenotypic traits and functional enrichment of key modules. (**A**) Heatmap of module–trait correlations. Each row represents a specific gene co-expression module, and each column represents a phenotypic trait (BCI: body condition index; FA: forearm length; HW: horseshoe width; SW: sella width; SH: sella height; RF: resting frequency). Each cell contains the Pearson correlation coefficient with the corresponding *p*-value in parentheses. The color scale indicates the strength and direction of the correlation. (**B**,**C**) Functional enrichment analyses of the Black and Purple modules, respectively. The y-axis displays the GO/KEGG term descriptions, and the x-axis represents the statistical significance as –log_10_(*p*-value). Bubble size corresponds to the number of genes enriched in each term. The top seven GO terms per category and the top 20 enriched KEGG pathways are displayed.

A total of 13 distinct co-expression modules were identified in WGCNA (Figure 3A). The Grey module, which contains genes that did not group with other modules, represented unclustered genes. Module-trait analysis showed that specific modules correlated significantly with echolocation frequency and morphological parameters. The Black module had a strong positive correlation with RF (*r* = 0.94, *p* < 0.001) and a significant negative correlation with HW (*r* = −0.93, *p* < 0.001). The Purple module showed a significant negative correlation with RF (*r* = −0.72, *p* < 0.05) and a positive correlation with noseleaf parameters (SW and SH).

Functional enrichment analysis of the Black module revealed many pathways related to aerobic respiration and rapid energy mobilization (Figure 3B, Tables S6 and S7). The top enriched GO terms included “Tricarboxylic acid cycle,” “Aerobic respiration,” and “Energy derivation by oxidation of organic compounds.” KEGG pathway analysis showed significant enrichment for “Citrate cycle (TCA cycle),” “Carbon metabolism,” and “Propanoate metabolism.” The Purple module had a different metabolic focus, centered on lipid metabolism (Figure 3C, Table S6 and S7). GO enrichment showed that genes in this module were associated with “Fatty acid beta-oxidation,” “Lipid modification,” and “Cellular lipid metabolic process.” KEGG analysis identified significant enrichment in “Fatty acid degradation,” “Fatty acid metabolism,” and the “PPAR signaling pathway.”

### 3.4. Identification and Evolutionary Conservation of the Hub Gene ACTC1

**Figure 4:**
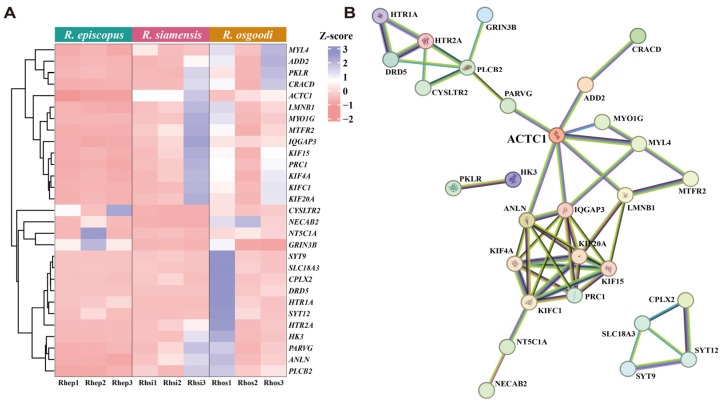
Expression profiles and protein–protein interaction (PPI) network of candidate hub genes. (**A**) Hierarchical clustering heatmap of 29 candidate differentially expressed genes across the three *Rhinolophus* species. Expression values are row-scaled (Z-score), with blue indicating relative upregulation and pink indicating relative downregulation. (**B**) The PPI network of the corresponding proteins, highlighting ACTC1 as a central hub interacting with cytoskeleton- and muscle-related proteins. Network nodes represent proteins, and edges represent functional associations predicted in STRING.

Twenty-nine candidate genes related to muscle contraction, cytoskeletal organization, and motor activity were prioritized from the identified DEGs. Hierarchical clustering revealed distinct expression patterns across the three species (Figure 4A). PPI analysis identified *ACTC1* as the central hub, with interactions involving cytoskeleton- and muscle-related proteins, such as MYL4, IQGAP3, and KIF family members (Figure 4B).

Phylogenetic analysis showed the *ACTC1* gene tree closely matches the species tree (Figure S5), indicating strong evolutionary conservation. The LRT found no significant evidence of positive selection on *ACTC1* (*p >* 0.05; Table S8), so the null hypothesis was not rejected.

## 4. Discussion

Understanding the genetic basis of complex phenotype diversification is a key challenge in evolutionary biology. While the ecological significance of bat echolocation is well-established [10,11,68], the molecular mechanisms regulating the laryngeal apparatus to produce distinct frequencies among species remain largely elusive. Here, we show that integrating differential expression analysis, functional enrichment analysis, co-expression network analysis, and molecular evolution analysis of laryngeal transcriptomic data reveals transcriptional signatures associated with differences in echolocation frequency and acoustic divergence across species.

### 4.1. Transcriptional Signatures of Laryngeal Contractile and Structural Adaptations

High-frequency echolocation imposes stringent biomechanical demands on the vocal folds. This requires the laryngeal muscles to generate rapid and precise contractions, a process physiologically dependent on superfast muscles [26]. Consistent with these structural requirements and the first hypothesis of our study, functional enrichment analyses revealed that upregulated genes in the high-frequency species (*R. osgoodi* and *R. siamensis*) were significantly enriched in the “Motor proteins” and “Cell adhesion molecules” pathways (Figure 2).

The enrichment of “Motor proteins” likely reflects the molecular basis for the high contraction speeds required by high-frequency callers [69]. Previous comparative genomic studies have demonstrated that genes encoding fast-twitch muscle fiber proteins are predominantly expressed in the superfast sound-producing muscles of echolocating bats [29]. A distinguishing feature of these superfast muscles is their high fiber shortening velocity, which is driven by myosin motors (e.g., *Myh2* and *Myl1*) with rapid cross-bridge detachment kinetics [29,70,71]. Complementing these genomic findings, our transcriptomic analysis revealed differential expression of additional myosin superfamily members (e.g., *Myl4*, *Myo1g*) in the laryngeal muscles of high-frequency species.

Furthermore, the enrichment of “Cell adhesion molecules” indicates a structural adaptation essential for superfast muscle function. These transmembrane proteins mediate cell-cell and cell-extracellular matrix (ECM) interactions, playing critical roles in skeletal muscle development and maintenance [72]. Specifically, they are required for myofibril anchoring, sarcomere alignment, and neuromuscular junction stabilization [73,74]. Their upregulation in high-frequency species suggests that extreme contraction frequencies require enhanced mechanical stability. This stability helps muscles withstand repetitive strain and prevents tissue damage. The co-adaptation of contractile and structural machinery highlights the multifaceted molecular basis of superfast vocal muscle evolution.

### 4.2. Energy Metabolism Trade-Offs Across Echolocation Frequencies

The availability of ATP is critical for skeletal muscle contractile activity [75]. As one of the most metabolically demanding tissues in vertebrates, superfast muscles require sustained and rapid ATP regeneration to maintain extreme contractile performance. A primary pathway for ATP regeneration is oxidative phosphorylation, which in mammals predominantly utilizes carbohydrates and lipids as substrates [76,77]. Notably, carbohydrate oxidation provides a higher ATP production rate, whereas fat oxidation offers a greater total ATP yield [75]. Consistent with this, exercise physiology studies demonstrate that carbohydrate oxidation dominates at higher exercise intensities, whereas fat oxidation is more important at lower intensities [75].

Expanding on these metabolic considerations and supporting our second hypothesis, the WGCNA analysis revealed divergent enrichment of substrate-specific metabolic pathways (Figure 3), which may reflect the established relationship between exercise intensity and substrate utilization. Specifically, high-frequency echolocation represents a high-intensity performance state for laryngeal muscles, imposing extreme metabolic demands due to the rapid rate of contraction. The module associated with high-frequency species (Black module) is characterized by upregulation of carbon and pyruvate metabolism, indicating a preference for carbohydrates to fuel these rapid muscle contractions. Furthermore, the concurrent enrichment of key pathways involved in aerobic energy metabolism (e.g., “Citrate cycle” and “Cellular respiration”) suggests a continuous, high-yield supply of ATP via oxidative phosphorylation.

In contrast to the high-frequency species, the module correlated with low-frequency species (Purple module) is predominantly enriched in “Fatty acid beta-oxidation,” “Fatty acid oxidation,” “Fatty acid degradation,” and the “PPAR signaling pathway” (Figure 3C), highlighting a primary reliance on lipid substrates. Compared to high-frequency bats, lower-frequency species, typically characterized by larger body mass, are metabolically predisposed to rely on lipids. While lipid oxidation proceeds at a relatively slow rate, it generates the highest yield of ATP per unit mass [78]. Consequently, this metabolic profile favors energetic efficiency over instantaneous power output, supporting both the long-term homeostatic maintenance and the prolonged endurance demands of larger bats.

Existing research on bat bioenergetics has predominantly focused on flight metabolism [79,80,81,82]. However, the relationship between echolocation and metabolic rate remains largely unexplored [80]. A recent study identified a significant positive correlation between basal metabolic rate and peak frequency of echolocation in most bat species [83]. Our transcriptomic findings offer a molecular perspective on this macrophysiological phenomenon, showing that different vocalization frequencies are associated with distinct energy supply mechanisms. This suggests that at the cellular level, closely related species with different echolocation frequencies may have evolved specific metabolic substrate preferences (e.g., carbohydrate versus lipid utilization) to help meet their unique energetic demands.

### 4.3. Regulatory Role and Evolutionary Constraints of ACTC1

Within the PPI network, *ACTC1* emerged as a highly connected hub, interacting with multiple proteins involved in muscle contraction and cytoskeletal organization (Figure 4B). The *ACTC1* gene encodes alpha-cardiac actin, a core component of sarcomeric thin filaments that provides the essential actin track for myosin-based force generation [84]. Although *ACTC1* is expressed in both skeletal and cardiac tissues, it accounts for ~80% of total actin in adult cardiac muscle and only ~5% in adult skeletal muscle [85]. Despite this low abundance in skeletal muscle, pathogenic variants in *ACTC1* are known to cause severe skeletal muscle contractures, highlighting its critical functional role [86,87].

Building upon this, our study validates the importance of *ACTC1* in laryngeal skeletal muscle at the transcriptional level. We found this gene to be differentially expressed among species with distinct vocalization frequencies (Figure 4A) and to be involved in the previously discussed critical “Motor proteins” pathway. Notably, the *ACTC1* gene is also present in the Black module identified by WGCNA, where it is co-expressed with genes related to energy metabolism pathways. These results imply that achieving high-frequency echolocation may require the tight transcriptional co-regulation of muscle contraction and metabolic energy supply.

Contrary to our third hypothesis, molecular evolutionary analysis shows that the *ACTC1* coding sequence is highly conserved among the closely related species analyzed, with no signals of positive selection detected (Table S8). This extreme conservation matches its fundamental role as a core structural protein in muscle contraction, where amino acid sequences are subject to strong purifying constraints [88]. Therefore, *ACTC1* likely contributes to echolocation frequency divergence by differentially regulating expression in laryngeal muscles or altering interactions with other contraction-related proteins, rather than through coding-sequence mutations. These findings support the idea that, among closely related species, regulatory evolution often plays a larger role than structural protein evolution in driving rapid divergence of complex phenotypes [89,90,91].

### 4.4. Limitations and Future Directions

While our analysis identified substantial transcriptomic divergence between the high- and low-frequency species, it is important to interpret these molecular insights in light of certain methodological constraints. Primarily, this study employed a modest sample size (*n* = 3 per species), a sampling strategy chosen to minimize the ecological impact on these wild bat populations. This constraint likely explains why the expression differences between the two high-frequency bats (*R. siamensis* and *R. osgoodi*) were less pronounced, as evidenced by their limited number of DEGs (Figure 1B) and overlapping PCA clusters (Figure S2A). Although algorithms like DESeq2 are specifically designed to robustly handle such sample sizes [55,92], a small sample size inherently limits the statistical power required to detect fine-scale, subtle transcriptomic variations. Similarly, this limitation may reduce the stability of module detection in WGCNA. Nevertheless, a previous study demonstrated that when robust phenotypic gradients are present, even small sample sizes can effectively capture key regulatory networks [93].

Furthermore, while de novo transcriptome assembly is a widely utilized and powerful tool for capturing species-specific transcripts, it can be complicated by challenges such as reduced transcript recovery and the generation of false-positive isoforms [94,95,96]. To prioritize a stable, reliably annotated structural scaffold for our differential expression analysis, we used the high-quality reference genome of the closely related *R. ferrumequinum*. Nevertheless, we acknowledge that this cross-species alignment strategy inevitably introduces bioinformatic limitations, including reduced mapping efficiency, potential annotation discrepancies, and the omission of species-specific transcripts. Finally, while our analyses reveal significant frequency-associated expression differences in the laryngeal tissues, it is crucial to recognize that transcript-level changes do not necessarily correspond directly to protein abundance or definitive functional outputs.

Therefore, our results should be viewed as testable hypotheses for future research. To fully elucidate the mechanisms underlying interspecific frequency divergence and capture those species-specific transcripts missed by cross-species alignment, future studies should employ larger sample sizes and de novo transcriptome assemblies to enhance analytical resolution. Ultimately, integrating transcriptomic data with evidence from multiple biological levels, such as detailed laryngeal morphoanatomy, targeted proteomics, and in vivo physiological assays, while extending these comparative analyses to include both sexes and a broader range of bat taxa, will yield deeper insights into the ecological and evolutionary patterns shaping acoustic adaptation in bats.

## 5. Conclusions

Through comparative transcriptomic profiling of laryngeal tissues across closely related *Rhinolophus* species, this study demonstrates that differences in echolocation frequency are tightly linked to laryngeal muscle contraction and energy supply, providing a crucial molecular basis for existing behavioral and acoustic observations. Furthermore, our findings highlight a potential regulatory mechanism by which differential gene expression fine-tunes metabolic substrate utilization to support this acoustic divergence. Specifically, our data indicate that high-frequency species upregulate pathways of aerobic respiration and carbon metabolism, whereas lower-frequency species rely more on lipid metabolism. Importantly, rather than relying on widespread coding-sequence mutations, the physiological differences underlying acoustic divergence may be achieved through gene expression regulation. This is exemplified by the highly conserved hub gene *ACTC1*, suggesting that altering expression levels can confer functional differences while avoiding potentially deleterious mutations in essential core proteins. While our transcriptomic data support clear regulatory differences in metabolic and contractile pathways within the bat larynx, the direct effects of these transcriptional changes on final protein levels and actual muscle performance remain to be determined. Future integrative studies combining detailed laryngeal morphoanatomical, targeted proteomic, and in vivo functional assays will be required to fully elucidate the physiological mechanisms and evolutionary significance of these molecular adaptations in echolocating bats.

## Data Availability

Data are contained within the article and Supplementary Materials. The data presented in this study are available upon request from the corresponding authors.

## Supplementary Materials

The following supporting information can be downloaded at https://www.mdpi.com/article/10.3390/biology15070548/s1: Figure S1. Correlation matrix of morphological traits and resting frequency (RF) among three *Rhinolophus* species. Figure S2. Global transcriptional divergence of laryngeal tissues among three *Rhinolophus* species. Figure S3. Dispersion estimation and empirical Bayes shrinkage of the laryngeal transcriptome. Figure S4. Gene set enrichment analysis (GSEA) of KEGG pathways across three pairwise comparisons. Figure S5. Species and gene trees used for evolutionary analyses. Table S1. Summary of sample information and sequencing data for three *Rhinolophus* species. Table S2. PCR primers and annealing temperatures used for *ACTC1* amplification in this study. Table S3. Taxonomy, echolocation capabilities, and sequence sources of the 27 mammalian species used for evolutionary analyses. Table S4. Detailed results of Gene Ontology (GO) enrichment analysis of differentially expressed genes (DEGs). Table S5. Detailed results of KEGG pathway enrichment analysis of differentially expressed genes (DEGs). Table S6. Detailed results of Gene Ontology (GO) enrichment analysis of genes from WGCNA modules. Table S7. Detailed results of KEGG pathway enrichment analysis of genes from WGCNA modules. Table S8. Results of branch-site model analyses for detecting positive selection on the *ACTC1* gene.
