# Supplementary material for: Laryngeal Transcriptomic Insights into Echolocation Call Frequency Divergence in Closely Related Rhinolophus Species
Source: Biology (Basel). 2026 Mar 30;15(7):548. doi: 10.3390/biology15070548 (PMC13072195; doi:10.3390/biology15070548)
Supplement: Supplementary file 1 [file biology-15-00548-s001.zip › Supplementary Figures.pdf]

Supplementary Materials

Laryngeal Transcriptomic Insights into Echolocation Call Frequency Divergence in Closely Related *Rhinolophus* Species

Guiyin Miao [1,2], Jinhua Cong [1,2], Jinhong Lei [1,2], Sirui Quan [3], Jiqian Li [4], Yannan Li [4], Kangkang Zhang [4,*] and Tong Liu [1,2,*]

[1] College of Life Sciences, Jilin Agricultural University, Changchun 130118, China
[2] Jilin Provincial International Cooperation Key Laboratory for Biological Control of Agricultural Pests, Changchun 130118, China
[3] College of Animal Science and Technology, Jilin Agricultural University, Changchun 130118, China
[4] Jilin Provincial Key Laboratory of Animal Resource and Ecological Security, Northeast Normal University, Changchun 130117,China
* Correspondence: zhangkk307@nenu.edu.cn (K.Z.); liut035@nenu.edu.cn (T.L.)

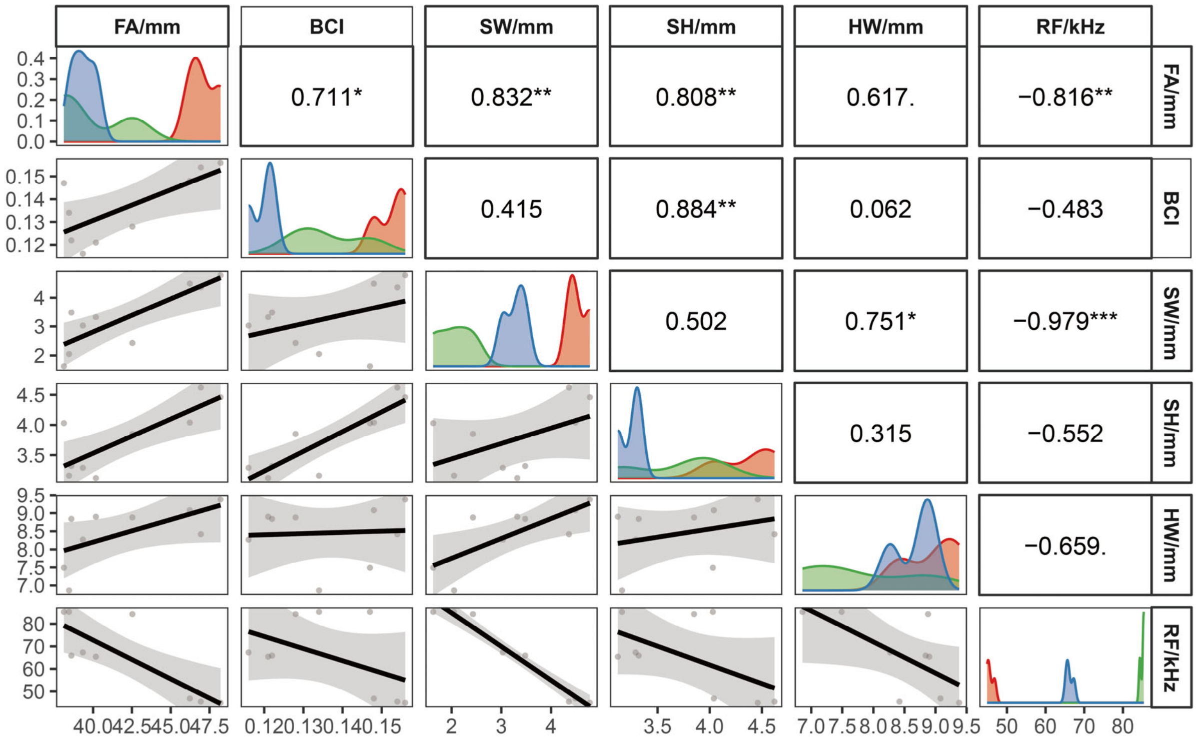

Figure S1. Correlation matrix of morphological traits and resting frequency (RF) among three *Rhinolophus* species. The diagonal plots display the kernel density distribution of each variable, with distinct colors representing different species (Red: Rhep; Blue: Rhsi; Green: Rhos). The lower triangular matrix shows the bivariate scatter plots with a global linear regression line (black solid line) and 95% confidence intervals (gray shading), illustrating the overall phenotypic trajectories across all studied individuals. The upper triangular matrix provides the overall Pearson correlation coefficients (r) for the pooled dataset. Significance levels are indicated by asterisks: *$p < 0.05$, **$p < 0.01$, ***$p < 0.001$. FA, forearm length; BCI, body condition index; SW, sella width; SH, sella height; HW, horse-shoe width.

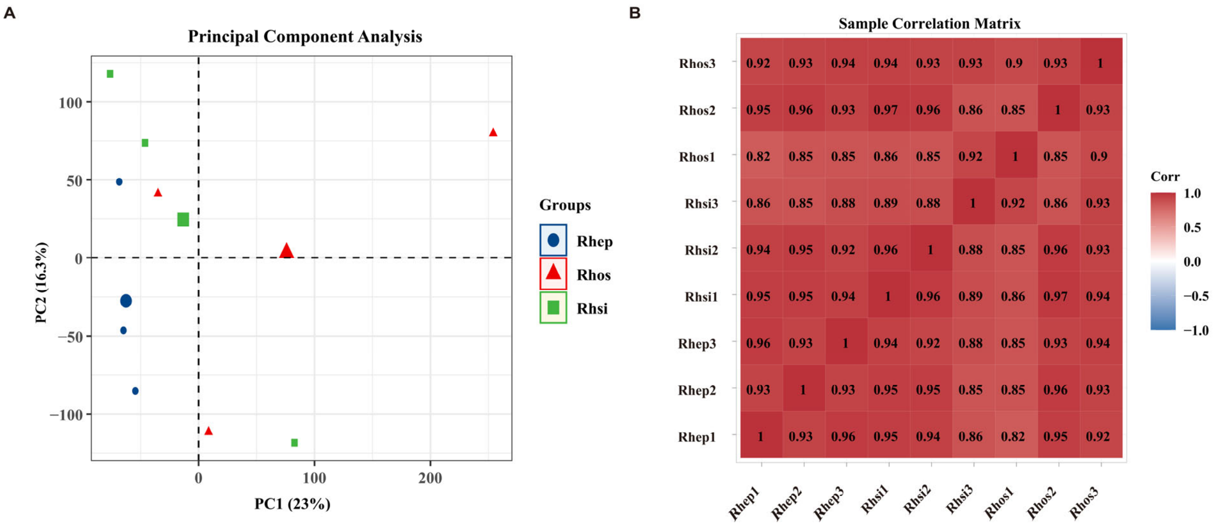

Figure S2. Global transcriptional divergence of laryngeal tissues among three *Rhinolophus* species. (A) Principal Component Analysis (PCA) plot. The first two principal components (PC1 and PC2) together account for 39.3% of the total variance (23.0% and 16.3%, respectively). Small symbols represent individual biological replicates, while the larger symbols indicate the group centroids for each species. This separation is consistent with the number of DEGs identified in pairwise comparisons (Figure 1B), confirming the distinct transcriptional profile of *R. episcopus*. **(B) Sample Pearson correlation heatmap.** Individual samples are color-coded in a matrix based on their pairwise Pearson correlation coefficients (r) calculated from gene expression profiles. The key indicates the correlation scale, with darker red representing stronger correlation. High correlations (r > 0.8) among biological replicates within the same species confirm data reliability.

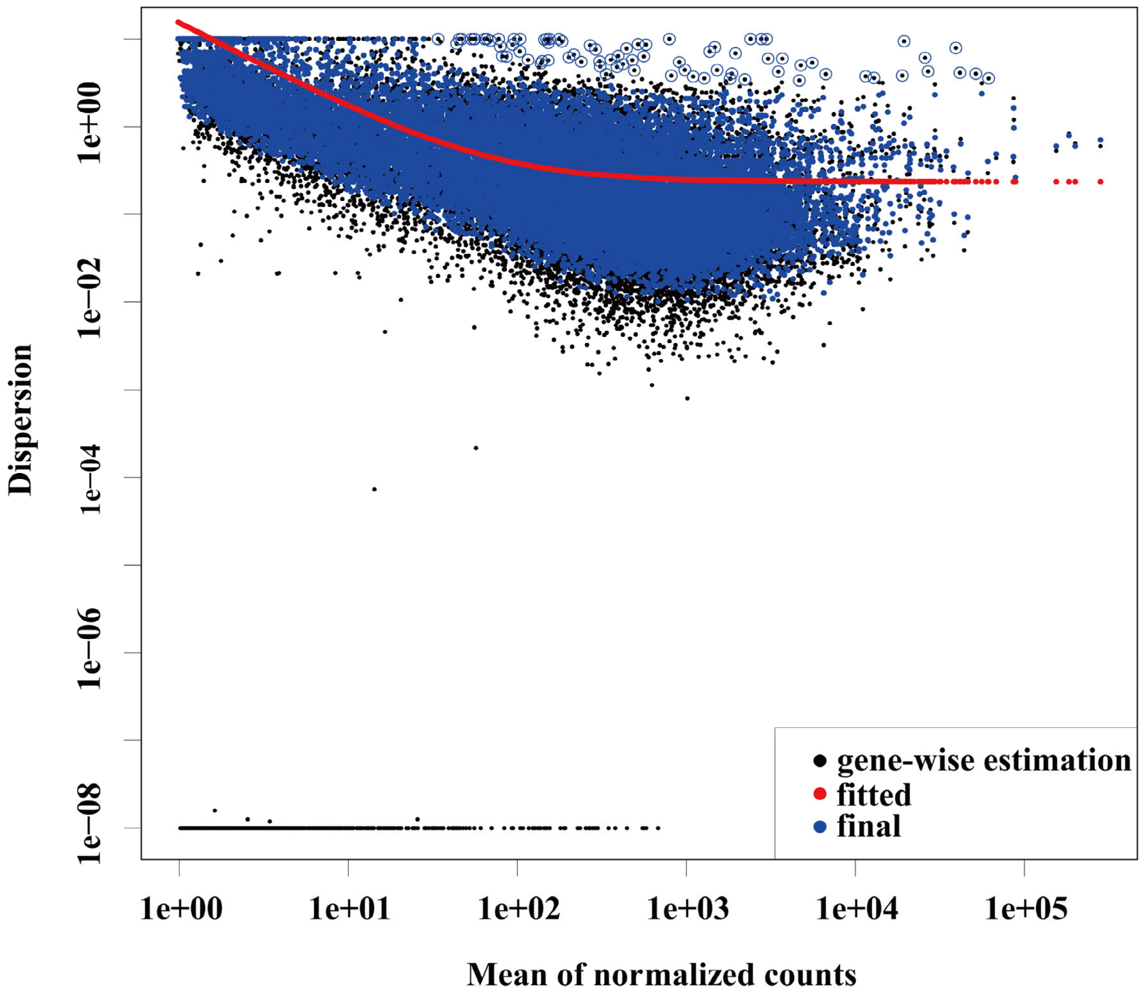

Figure S3. Dispersion estimation and empirical Bayes shrinkage of the laryngeal transcriptome. A plot for assessing the variance-mean relationship across the transcriptome. Black dots represent gene-wise dispersion estimates; the red line shows the fitted mean-dispersion trend; and blue dots indicate the final dispersion values shrunken towards the trend. The characteristic distribution (downward curve and subsequent stabilization) demonstrates the inherent structure of biological variation in our datasets and confirms the high quality of the raw sequence counts for subsequent statistical modeling.

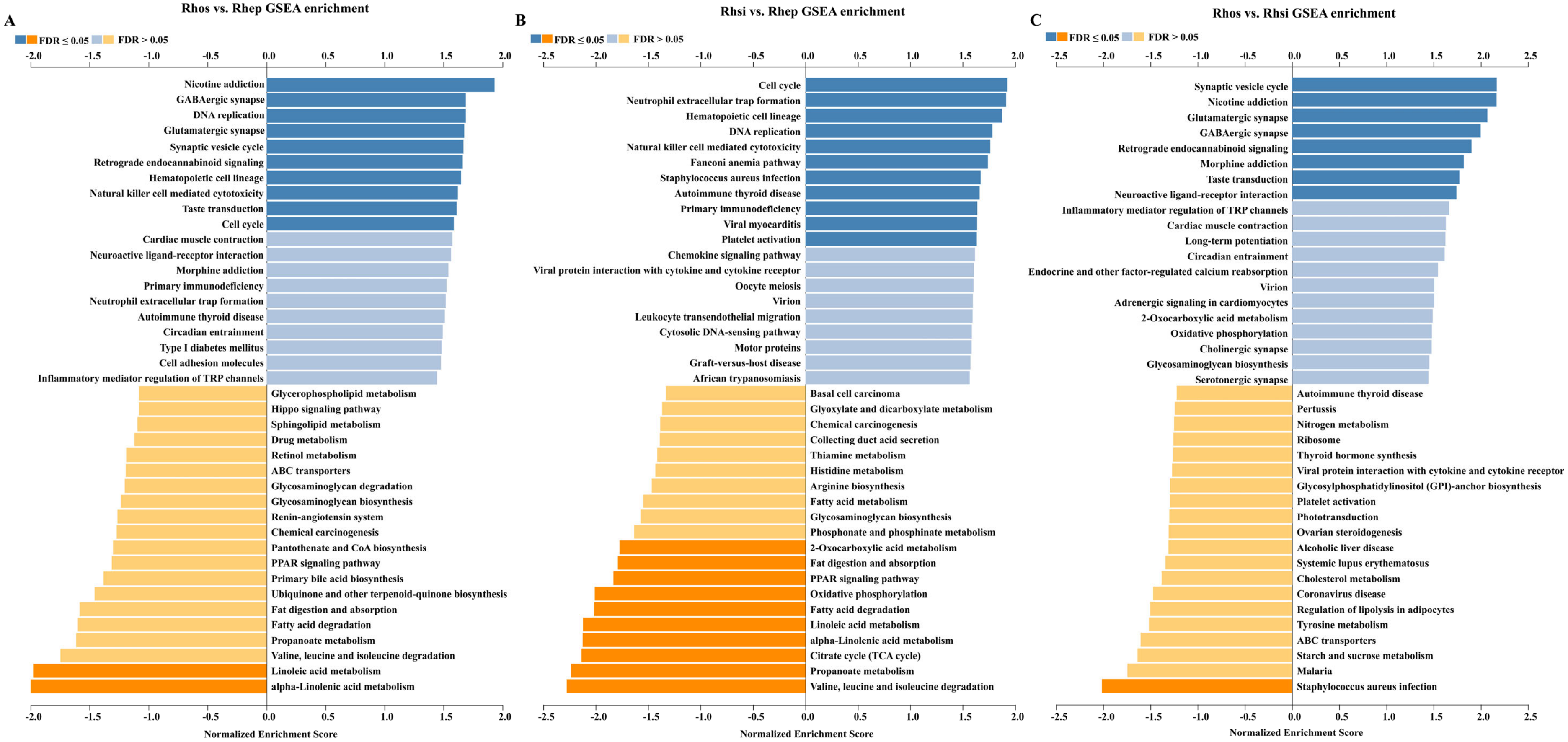

Figure S4. Gene set enrichment analysis (GSEA) of KEGG pathways across three pairwise comparisons. (A) Rhos vs. Rhep. **(B)** Rhsi vs. Rhep. **(C)** Rhos vs. Rhsi. The x-axis represents the Normalized Enrichment Score. Blue bars indicate pathways enriched in the first species of each comparison. Orange bars indicate pathways enriched in the second species. Color intensity corresponds to statistical significance. Darker colors represent significant enrichment with a False Discovery Rate (FDR) ≤ 0.05, whereas lighter colors indicate FDR > 0.05 and *p*-value ≤ 0.05.

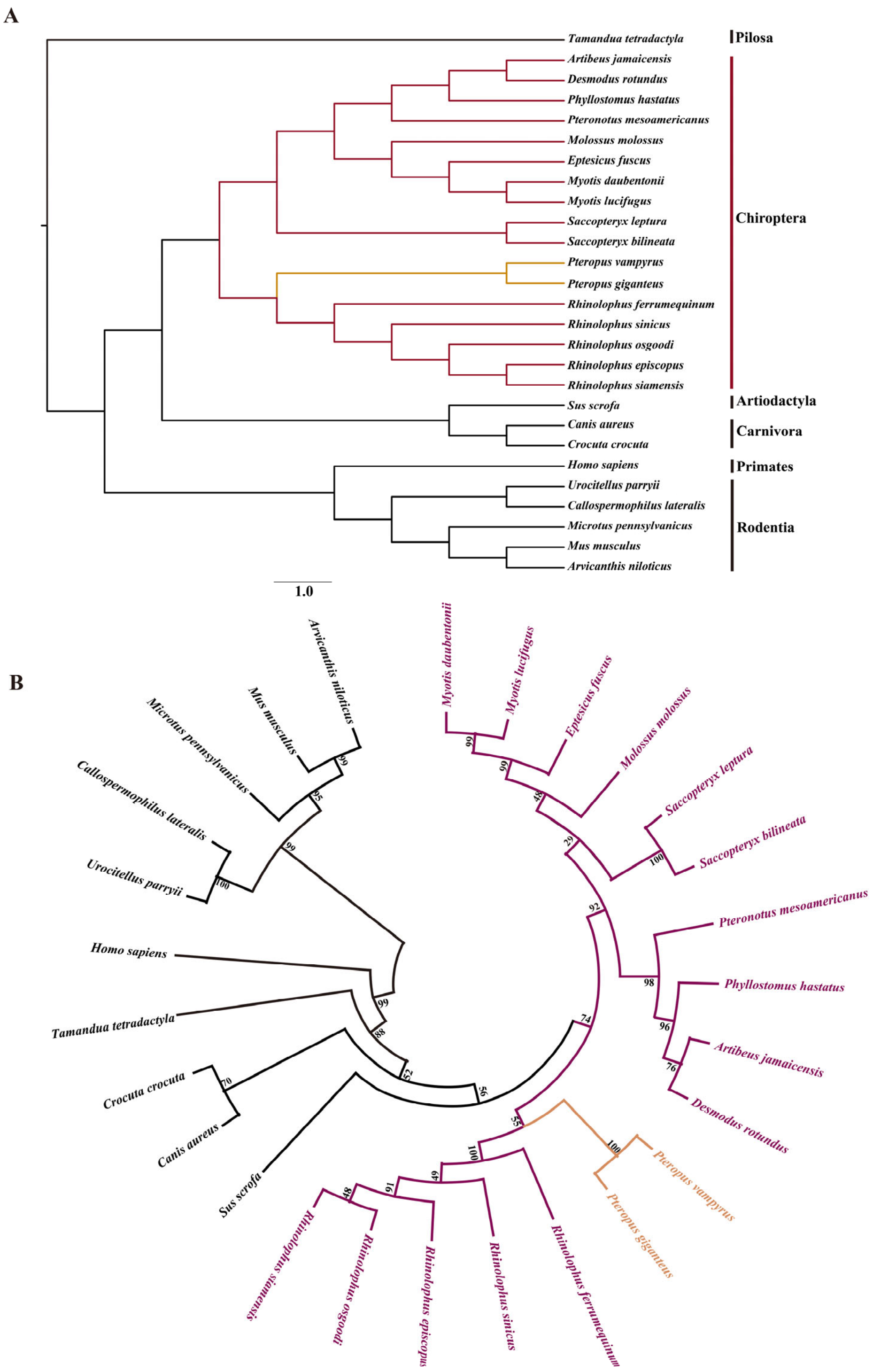

Figure S5. Species and gene trees used for evolutionary analyses. (A) Species tree inferred from 27 mammalian species. **(B)** Gene tree reconstructed from the coding sequence (CDS) of *ACTC1* across the same 27 species. Lineages of echolocating bats are highlighted in purple-red, whereas non-echolocating bats are highlighted in orange. Gene tree was constructed using the Maximum Likelihood method with 1000 bootstrap replicates.
